# The landscape of cocaine cytotoxicity and the role of sigma-1 receptor modulation and adulterant synergism

**DOI:** 10.1007/s00204-026-04434-8

**Published:** 2026-05-13

**Authors:** Aline Steinmetz, Carlo Frederico Moro, Luana Freese, Murilo Sander de Abreu, Rodrigo Ligabue Braum, Helena Maria Tannhauser Barros, Dinara Jaqueline Moura

**Affiliations:** 1 Graduate Program in Pathology, Federal University of Health Sciences of Porto Alegre, Porto Alegre, RS Brazil; 2 Laboratory of Genetic Toxicology, Federal University of Health Sciences of Porto Alegre, Sarmento Leite 245, Sala 714, Prédio 3, Centro Histórico, Porto Alegre, RS 90050-170 Brazil; 3 Graduate Program in Biosciences, Federal University of Health Sciences of Porto Alegre, Porto Alegre, RS Brazil; 4 Laboratory of Neuropharmacology, Federal University of Health Sciences of Porto Alegre, Porto Alegre, RS Brazil; 5 Graduate Program in Health Sciences, Federal University of Health Sciences of Porto Alegre, Porto Alegre, Brazil

**Keywords:** Cocaine, Toxicity, Oxidative stress, Adulterants, Sigma receptor

## Abstract

Cocaine remains one of the most widely consumed illicit drugs globally, representing a significant public health challenge. While its acute reinforcing effects are mediated by the facilitation of dopaminergic and serotonergic neurotransmission, chronic exposure leads to pervasive neurobiological adaptations and systemic toxicity. Beyond its psychoactive properties, cocaine exerts multifaceted cytotoxic effects across several organ systems, including the brain, heart, and liver, primarily through the induction of oxidative stress, mitochondrial dysfunction, and the activation of apoptotic pathways. This review provides a comprehensive analysis of these cellular and molecular mechanisms and introduces novel evidence regarding the toxicological impact of seized cocaine. Original in vitro data demonstrate that the association of cocaine with common adulterants, levamisole, phenacetin, and caffeine, markedly exacerbates cytotoxicity through synergistic interactions. Furthermore, this review examines the pivotal role of the sigma-1 receptor (σ1R) in cocaine-induced toxicity, supported by molecular docking analyses that characterize specific interactions between the cocaine, adulterants, and conserved receptor residues. This receptor-mediated framework suggests a central mechanism contributing to the drug’s combined toxic and reinforcing properties. Collectively, these findings integrate experimental, computational, and literature-based evidence to offer a broader mechanistic understanding of cocaine-induced toxicity and its modulation by adulterants, providing compelling evidence for the role of σ1R in these cytotoxic processes.

**Supplementary Information:**

The online version contains supplementary material available at 10.1007/s00204-026-04434-8.

## Introduction

Cocaine remains one of the most widely used illicit drugs worldwide and continues to pose a major public health concern. According to the World Drug Report 2025, global cocaine use has steadily increased over the past decade, reaching an estimated 25 million users in 2023, up from 17 million in 2013. This rise corresponds to an increase in prevalence from 0.36 to 0.47% among people aged 15 to 64. In parallel, global cocaine seizures reached a record high in 2023, with a 68% increase in the quantity seized between 2019 and 2023. The largest markets remain North America, Western and Central Europe, and South America, based on both user estimates and wastewater analysis data (UNODC 2025; Steinmetz 2017).

Cocaine produces its psychoactive and addictive effects primarily by acting on the brain’s reward system, a set of interconnected regions that regulate pleasure and motivation (Nestler 200). Cocaine binds to and inhibits the reuptake of the monoamine neurotransmitters dopamine, norepinephrine and serotonin by blocking their respective transporters (DAT, NET, and SERT) with comparable affinity. However, its predominant psychostimulant and reinforcing effects arise mainly from DAT blockade, which markedly increases extracellular dopamine levels in the striatum (Trifilieff and Martinez 2014). Monoamines accumulate in the synaptic cleft, resulting in enhanced and prolonged sympathetic effects; while the build-up of dopamine drives euphoria and addiction (Ritz et al. 1987), the concomitant catecholaminergic surge triggers systemic physiological stress.

Beyond these neuroadaptive changes, cocaine addiction is rapidly progressive and associated with severe medical consequences that transcend its psychotropic properties (Cregler 1989). Emerging evidence underscores that these clinical complications are deeply rooted in the drug’s inherent cytotoxicity. The metabolic degradation of cocaine and the resulting monoaminergic imbalance catalyze a cascade of oxidative stress, mitochondrial dysfunction, and programmed cell death (Badisa et al. 2018; Donnelly et al. 1988; Liou et al. 2014; Dietrich et al. 2005). This toxicological landscape is not restricted to the central nervous system but extends to cardiovascular, hepatic, and renal tissues, where the drug’s detrimental effects are frequently exacerbated by the presence of pharmacologically active adulterants (Acosta and Anuforo 1976; Kazaks et al. 2019; Patierno et al. 1989).

In addition to its well-established action on monoamine transporters, the activation of sigma-1 receptors (σ1R) represents a critical molecular component associated with the behavioral and toxic effects of cocaine (Navarro et al. 2010). While the transporter-mediated catecholaminergic surge initiates systemic stress, σ1R acts as a multifaceted chaperone, modulating the intracellular environment in response to the drug. This receptor serves as a pivotal link between the initial neurochemical imbalance and the subsequent cellular damage, influencing processes such as calcium homeostasis, endoplasmic reticulum stress, and mitochondrial integrity (Lever et al. 2016). Consequently, the interaction between cocaine and σ1R constitutes a fundamental mechanism in the drug’s multisystemic cytotoxicity and neuroadaptive outcomes.

Recognizing the growing relevance of cocaine-induced systemic injury, this review first delineates the canonical cellular and molecular mechanisms underlying the drug’s cytotoxicity, drawing upon established evidence from in vitro and in vivo models. Subsequently, the scope is expanded to integrate novel evidence regarding the cytotoxic impact of common adulterants, specifically phenacetin, levamisole, and caffeine, highlighting how their synergistic interactions exacerbate cellular damage. Finally, the role of the sigma-1 receptor σ1R as a pivotal mediator that orchestrates these cytotoxic processes is addressed. By combining traditional mechanistic insights with original in vitro and in silico data, this review provides a comprehensive and updated landscape of the toxicological reality of seized cocaine.

### The systemic landscape of cocaine-induced cytotoxicity

Studies both in vivo and in vitro have consistently demonstrated that exposure to cocaine and its primary metabolites induces significant cytotoxic effects across multiple biological systems. In the Central Nervous System (CNS), cocaine exposure has been shown to profoundly affect neurons and glial cells; in murine models, this is characterized by a dramatic initial reduction in neurite number and length, followed by extensive neuronal death (Nassogne et al. 1995). These findings are further supported by in vitro models using NG108—15 and C6 cell lines, where exposure to benzoylecgonine, the major metabolite of cocaine, resulted in a loss of cell viability and detachment from the growth surface, confirming its inherent cytotoxicity for both neuronal and glial populations (Lin and Leskawa 1994).

The pathophysiology of these lesions in the CNS is deeply rooted in oxidative stress and redox imbalance. Acute and chronic cocaine administration has been shown to stimulate reactive oxygen species (ROS) production within dopaminergic brain structures, such as the frontal cortex and striatum of male rats, highlighting its oxidative damage potential (Dietrich et al. 2005). At the cellular level, this is reflected by an increase in malondialdehyde (MDA) production and a compensatory rise in superoxide dismutase (SOD) and catalase (CAT) activities. In C6 glioma cells, this oxidative response contributes to mitochondrial dysfunction and significant DNA damage (Steinmetz et al. 2018). Such genotoxic effects are not localized, as evidenced by increased DNA damage observed in the prefrontal cortex, hippocampus, striatum, and cerebellum of female rats following cocaine administration (de Souza et al. 2014).

The detrimental effects of cocaine extend to the cardiovascular system, where toxicity is driven by both functional impairment and programmed cell death. In humans, cocaine-related overdoses are associated with myocardial oxidative damage, largely induced by the activation of cardiac Fas-dependent and mitochondria-dependent apoptotic pathways (Turillazzi et al. 2017). Similar mechanisms have been identified in male rats, where chronic exposure triggers cardiomyocyte apoptosis through these same pathways (Liou et al. 2014). Furthermore, chronic exposure perturbs the antioxidant defense system, evidenced by elevated levels of SOD, glutathione peroxidase (GSH-Px), glutathione reductase (GR), and ascorbic acid (AA), alongside a critical depletion of glutathione (GSH) (Fineschi et al. 2001). Beyond direct cytotoxicity, in vitro data indicate that cocaine impairs cardiac contractility by inhibiting norepinephrine reuptake, enhancing vasoconstriction, and modulating ion channel activity (Fineschi et al. 2001).

Hepatic and renal tissues also exhibit high vulnerability to cocaine-induced redox disruption. In DBA/2Ha mice, acute exposure results in severe hepatotoxicity, characterized by mitochondrial membrane disruption, swelling, and endoplasmic reticulum stress (Gottfried et al. 1986). These morphological changes coincide with increased manganese-SOD (Mn/SOD) activity and thiobarbituric acid reactive substances (TBARS), while GSH-Px and CAT activities are diminished (Gottfried et al. 1986). This shift leads to heightened mitochondrial ROS production and a concomitant decrease in cellular ATP and GSH levels (Devi and Chan 1996). In vitro, cocaine and its metabolites, norcocaine and N-hydroxynorcocaine, further deplete hepatic glutathione, elevate hydrogen peroxide levels, and trigger the release of cytochrome c and caspase-3 activity (Thompson et al. 1979; Zaragoza et al. 2001). Similarly, cocaine and norcocaine exert nephrotoxic effects by reducing renal cell viability in a dose-dependent manner and compromising the antioxidant capacity of the glutathione system (Valente et al. 2012).

Cocaine promotes systemic toxicity through a complex interaction between immunosuppression and oxidative stress, with prolonged exposure exacerbating redox imbalance. Evidence from rat models (Pacifici et al. 2003) reveals that acute cocaine administration triggers a transient suppression of T-lymphocyte proliferation and natural killer (NK) cell activity, alongside a rapid shift from Th1 to Th2 cytokine profiles. These acute immunological shifts are accompanied by an immediate, albeit temporary, alteration in the antioxidant profile, characterized by an increase in AA, GSH, and GR activity.

These systemic alterations in redox homeostasis and immune function provide a broader physiological context for the specialized cellular damage observed in neural tissues. For instance, beyond direct redox imbalance, cocaine acts as a potent modulator of neuroinflammatory signaling and cellular plasticity. Chronic administration of the drug has been shown to increase the activity of NF-κB in the cerebellum of rats, a transcription factor widely recognized as a pivotal sensor of both oxidative stress and inflammatory responses (López-Pedrajas et al. 2015). This inflammatory cascade extends to the neurovascular unit, for instance, the exposure of pericytes to cocaine results in the significant upregulation of pro-inflammatory cytokines, including TNF-α, IL-1β, and IL-6, in both intra- and extracellular compartments, alongside an increased formation of autophagosomes (Sil et al. 2016).

Furthermore, cocaine disrupts the functional dynamics of neural progenitor cells (NPCs). Research indicates that cocaine inhibits the migratory response of these cells to CXCL12 (stromal cell-derived factor-1alpha), a phenomenon correlated with the drug-induced down-regulation of the CXCR4 receptor on the NPC surface. Additionally, NPCs exposed to cocaine undergo premature differentiation into cells expressing neuronal markers. This process is associated with the inhibition of SOX2 (SRY-related HMG-box gene 2), a transcription factor essential for maintaining the undifferentiated state of the progenitor pool (Xiao and Zhang 2008).

The cytotoxic reach of cocaine is further evidenced by its capacity to trigger programmed cell death through both intrinsic and extrinsic pathways, particularly during critical developmental stages. In the fetal brain, prenatal cocaine exposure has been shown to dose-dependently increase the activities of caspase-3, caspase-8, and caspase-9. This pro-apoptotic environment is driven by a significant modulation of the Bcl-2 family proteins, where the up-regulation of the Bax/Bcl-2 ratio serves as a primary mediator of cocaine-induced apoptosis (Xiao and Zhang 2008).

Consistent with these neurodevelopmental impacts, cocaine exerts profound apoptotic effects on myocardial tissue. In cultured fetal rat cardiomyocytes, the drug induces apoptosis in a time- and concentration-dependent manner, characterized by the mitochondrial release of cytochrome c and subsequent activation of the caspase-9 and caspase-3 cascade (Xiao et al. 2000). These molecular events are accompanied by severe structural and ultrastructural damage to myocardial cells (Welder et al. 1988, 1993). Furthermore, the metabolic signature of this toxicity includes marked ATP depletion and increased lactate dehydrogenase (LDH) leakage, indicating significant membrane injury and mitochondrial dysfunction—hallmarks that align with the activation of intrinsic apoptotic pathways (Yuan and Acosta 1996; Li et al. 2005).

The complexity of cocaine-induced cell death is also reflected in adrenal phaeochromocytoma (PC12) cell models. In NGF-differentiated PC12 cells, cocaine reduces viability and induces apoptosis by upregulating caspase-3 and -9 at concentrations up to 500 µM; notably, higher doses may shift the cellular response, failing to elicit the same apoptotic markers (Imam et al. 2012). In addition to apoptosis, cocaine triggers necrotic pathways in PC12 cells, evidenced by increased LDH release, α-spectrin cleavage, and down-regulation of the anti-apoptotic protein Bcl-2 (Lepsch et al. 2009). A critical mechanism in this toxicity is the opening of the mitochondrial permeability transition pore (PTP). Research has demonstrated that cocaine-induced death in these lineages is contingent upon PTP sensitization, as the administration of PTP inhibitors—such as cyclosporin A or metformin—significantly mitigates the cytotoxic effects of the drug (Lamarche et al. 2017).

The disruption of cellular ontogeny is further compounded by specific molecular checkpoints. As previously noted, cocaine exposure inhibits the proliferation of human fetal brain-derived neural precursor cells (NPCs) by inducing cell cycle arrest, a process likely mediated by the up-regulation of the cyclin-dependent kinase inhibitor p21 (Hu et al. 2006).

Beyond apoptosis and necrosis, cocaine has been shown to elicit autophagic cytotoxicity through a specialized signaling cascade involving nitric oxide and glyceraldehyde-3-phosphate dehydrogenase (GAPDH). This autophagic drive is characterized by a marked increase in LC3-II levels and the depletion of p62. Crucial evidence for this mechanism lies in the fact that pharmacological inhibition of autophagy, specifically the depletion of key mediators such as ATG5 or beclin-1, confers neuroprotection against cocaine-induced death, reinforcing the role of dysregulated autophagy in CNS pathology (Xiao and Zhang 2008).

In addition to these acute cellular insults, cocaine exposure exerts a profound influence on the epigenetic homeostasis of the cell, suggesting a potential for transgenerational toxicity. In male mice germ cells, the drug significantly perturbs the expression of various epigenetic regulators, evidenced by decreased levels of histone deacetylases (*Hdac1/2/8*), DNA methyltransferase *Dnmt3b*, and the ten-eleven translocation protein *Tet1*, alongside a concomitant increase in *Dnmt3a* expression (González et al. 2018). These epigenetic shifts indicate that cocaine-induced damage transcends immediate cytotoxicity, potentially altering the regulatory landscape of the genome in reproductive tissues.

In summary, the body of evidence underscores a multifaceted cytotoxicity of cocaine, primarily orchestrated through a complex interplay of redox imbalance, mitochondrial dysfunction, pro-apoptotic signaling, and epigenetic disruption. These deleterious effects are pervasive across diverse biological systems, establishing a robust framework for understanding how the cocaine independently compromises cellular integrity.

### Cocaine-adulterant interactions: expanding the landscape of cellular injury

Expanding upon these systemic impacts, it is crucial to recognize that several components involved in cocaine processing and adulteration exhibit significant cytotoxic potential. These include chemical residues from processing, such as solvents and alkalizing agents, and inert diluents like talc and boric acid. Furthermore, pharmacologically active adulterants, including caffeine, lidocaine, phenacetin, and levamisole, are frequently identified in seized samples and are known to potentiate the drug’s toxic and sympathomimetic effects (Lapachinske et al. 2015; Oliveira and Wagner 2013; Tallarida et al. 2014). For instance, exposure to caffeine (20 mM) has been shown to induce marked toxicity in rat heart cells, characterized by reduced viability, increased vacuolization, and pseudopod formation (Acosta and Anuforo 1976; Kazaks and Collier 2019). Likewise, phenacetin has been demonstrated to induce cytotoxicity in mouse embryo cell models (Patierno et al. 1989).

**Fig. 1:**
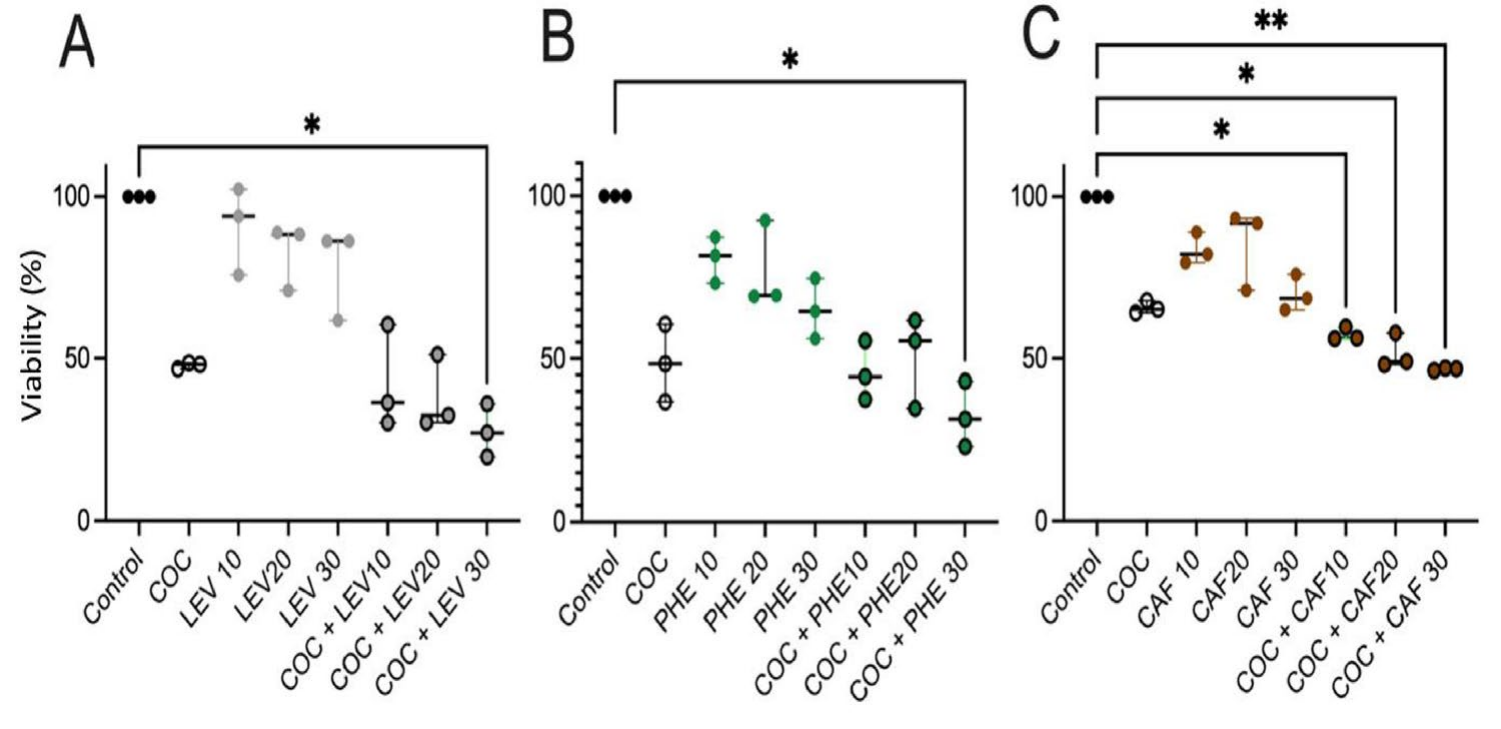
Cytotoxicity effects of cocaine and adulterants (levamisole (**A**), phenacetin (**B**) and caffeine (**C**) measured by MTT assay. Statistical analyses were performed using GraphPad Prism9 software (GraphPad Software, Boston, USA) and the Kruskal–Wallis test with *post-hoc* Dunn’s test. At least three independent experiments were pooled and analyzed as a combined data set. Data are expressed as median with interquartile range, with P set at < 0.05 in all analyses. Significant difference as compared to negative control treatment at *p < 0.05; and **p < 0.01. H_2_O was used as control. Results are detailed in Table 1 and Supplementary

To further elucidate how these associations impact cellular integrity, this review presents novel, unpublished data evaluating cell viability via the MTT assay under an acute exposure scenario. Following the experimental protocols and standardized conditions established in previous studies by the research group (Steinmetz 2017), the effects of cocaine (10 mM) were assessed both in isolation and in combination with common adulterants (levamisole, caffeine, and phenacetin) using a C6 rat glioma cell line (detailed methods available in Supplementary Information). In these experiments, the adulterant concentrations were fixed at 10, 20, and 30% relative to the cocaine, equivalent to 1, 2, and 3 mM of each compound, respectively. Statistical analysis, conducted via the Kruskal–Wallis test followed by Dunn’s post-hoc test, revealed that cocaine in isolation did not significantly compromise cell viability compared to the control group (Fig. 1 and Table 1). Similarly, treatment with the adulterants in isolation, at the concentrations tested, failed to reach statistically significant levels of cytotoxicity (Table 1).

**Table 1:** Statistical comparison between control group and test groups in MTT viability assay, showing Mean Rank Difference and *p* values according to Kruskal–Wallis test with *post-hoc* Dunn’s test

	Caffeine		Phenacetin		Levamisole	
	KW	*p*	KW	*p*	KW	*p*
Cocaine	11.33	0.3461	15.00	0.2608	13.00	0.6773
10% Cont	5.000	> 0.9999	4.333	> 0.9999	2.333	> 0.9999
20% Cont	4.333	> 0.9999	5.667	> 0.9999	5.000	> 0.9999
30% Cont	9.333	0.7397	8.667	> 0.9999	6.667	> 0.9999
Cocaine + 10% Cont	15.67	**0.0463**	16.00	0.1552	14.67	0.3076
Cocaine + 20% Cont	17.33	**0.0186**	14.33	0.3630	15.33	0.2196
Cocaine + 30% Cont	21.00	**0.0019**	20.00	**0.0147**	19.00	**0.0276**

Statistically significant results (*p* < 0.05) are in bold type

In contrast, a potent synergistic effect was observed when these substances were combined, particularly at higher concentrations. The association of cocaine with caffeine demonstrated a significant decrease in C6 cell viability across all tested concentrations (*p* < 0.05). For phenacetin and levamisole, significant cytotoxicity was primarily observed at the highest concentration of 30% (3 mM), yielding p-values of 0.0147 and 0.0276, respectively (Fig. 1, Table 1). These findings are pivotal, as they underscore that the high cytotoxic potential observed in seized cocaine is not merely an intrinsic property of the alkaloid, but is critically amplified by the synergistic presence of adjuvants and adulterants used in its illicit production.

### Sigma receptors and cocaine cytotoxicity

A growing body of evidence demonstrates that sigma receptors, particularly the σ1R subtype, are key modulators of the toxic and reinforcing properties of cocaine. Notably, cocaine binds directly to σ1R and can occupy these receptors in vivo at an ED_50_ approximately 2.5 times higher than that required for dopamine transporter (DAT) binding (Lever et al. 2017). This interaction has been shown to underlie critical behavioral outcomes, such as hyperlocomotion, convulsions, and lethality. In rodent models, the reduction of σ1R brain concentrations or the use of selective antagonists (e.g., BD1008, BD1047, and LR172) consistently attenuates cocaine-induced behavioral toxicity, whereas σ1R agonists exacerbate these responses (Lever et al. 2017; Navarro et al. 2010; McCracken, et al. 1999; Matsumoto et al. 2001).

At the cellular level, σ1R is broadly expressed in the central nervous system and peripheral tissues, where it exerts pleiotropic functions through its unique localization at the interface between the endoplasmic reticulum (ER) and mitochondria, known as the mitochondrial-associated membrane (MAM) (Novakova et al. 1995; Wolfe et al. 1997; Alonso et al. 2000; Maurice and Su 2009). Acting as a molecular chaperone, σ1R regulates ER stress responses, calcium homeostasis, mitochondrial metabolism, and apoptotic signaling pathways (Hayashi and Su 2003; Mavlyutov et al. 2017; Yasui and Su 2016). Cocaine, as an agonist, promotes σ1R oligomerization and dissociation from the chaperone BiP, enabling functional modulation of client proteins such as IP₃ receptors, ion channels, and dopamine transporters, thereby influencing both intracellular calcium flux and dopaminergic neurotransmission (Balasuriya et al. 2014; Nguyen et al. 2017; Chu and Ruoho 2016; Chu et al. 2013; Hayashi and Su 2007). In contrast, σ1R antagonists stabilize its association with BiP and suppress these downstream effects (Aydar et al. 2002).

Furthermore, the neuroinflammatory effects associated with cocaine appear to be intrinsically linked to sigma receptor activity. Research has demonstrated that cocaine-mediated activation of pericytes involves the dysregulation of autophagy and the upstream activation of ER stress pathways. This cascade results in the downstream production of pro-inflammatory cytokines, a process that is effectively abrogated by the pharmacological blocking of σ1R (Sil et al. 2019). These findings indicate that targeting σ1R or its upstream ER stress mediators represents a promising therapeutic strategy for mitigating cocaine-mediated inflammation in the neurovascular unit. Complementing these mechanisms, σ2R also contributes to the drug’s cytotoxic profile by regulating calcium signaling and promoting caspase-independent cell death, further establishing sigma receptors as central modulators of the cellular and behavioral consequences of cocaine exposure (Crawford and Bowen 2002; Hanner et al. 1996; Bowen 2000; Matsumoto and Mack 2001; Foster et al. 2003; Matsumoto et al. 2007).

**Fig. 2:**
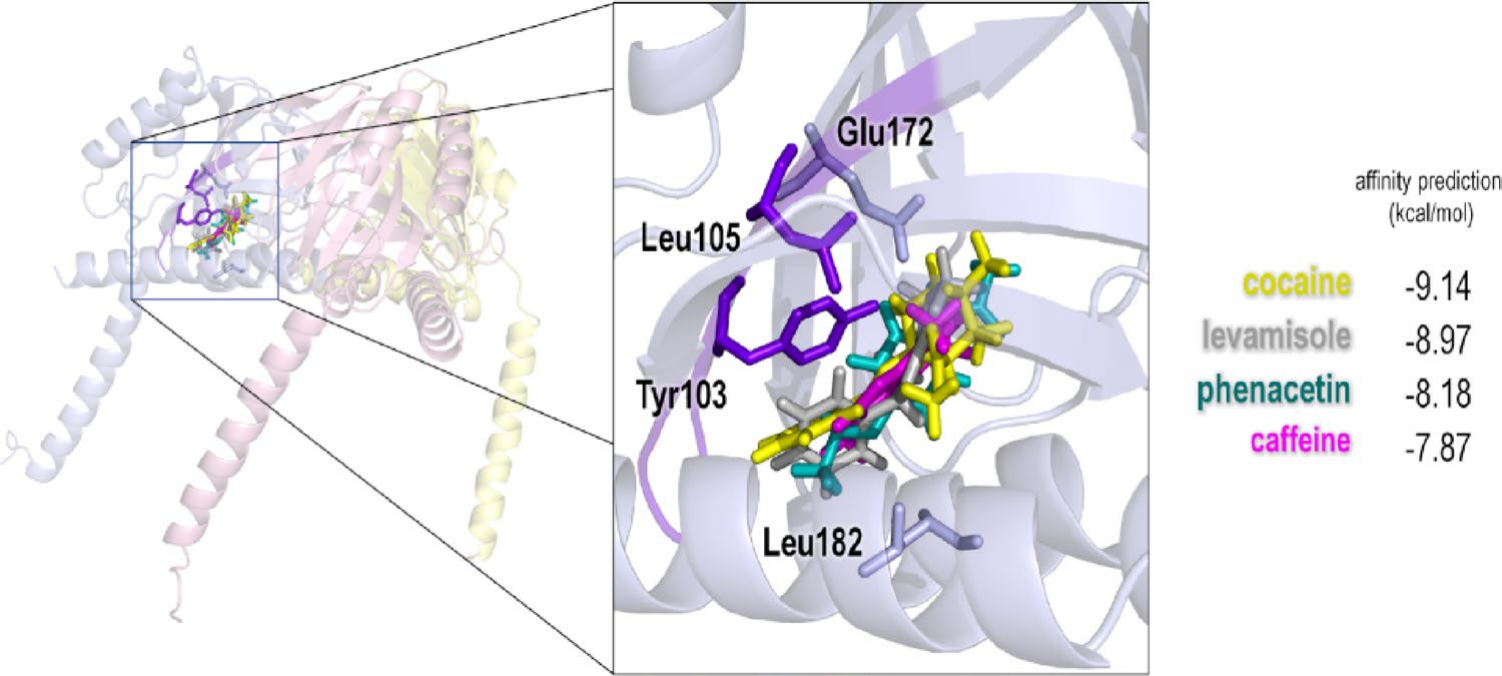
In silico molecular docking of cocaine and common contaminants with the σ1R. Conserved interactions were observed between all tested ligands and key σ1R residues, including Tyr103, Leu105, Glu172 (conserved ligand binding region, purple), and Leu182. Cocaine (yellow) exhibited the highest binding affinity (− 9.14 kcal/mol), followed by levamisole (grey) (− 8.97 kcal/mol), with phenacetin (green) (− 8.18 kcal/mol) and caffeine (pink) (− 7.87 kcal/mol) showing lower, but relevant, affinities

The molecular evidence implicating σ1R as a central target is further substantiated by novel in silico analyses conducted specifically for this review (Fig. 2). Recent advances in molecular docking protocols (Guedes et al. 2024) were employed to characterize the binding landscape of cocaine and its common adulterants within the σ1R binding pocket (detailed methods available in Supplementary Information). These computational assessments revealed highly conserved interactions between all tested ligands and specific residues of the receptor, with Tyr103, Leu105, Glu172, and Leu182 emerging as pivotal points of contact. Among these, Leu182 did not interact with caffeine, the smallest molecule tested, highlighting a possible steric or structural limitation in its binding mode. Tyr103 and Leu105 are located within the canonical ligand-binding region of σ1R, as annotated in UniProt (Q99720), while Glu172 is described as an essential residue for ligand interaction based on similarity to other crystallographic complexes (Schmidt et al. 2016, 2018). These results not only support the critical role of these residues in stabilizing cocaine binding, but also indicate that structurally related contaminants such as levamisole and phenacetin exploit the same interaction hotspots. The calculated binding energies corroborate this profile, with cocaine (− 9.14 kcal/mol) showing the highest affinity, followed closely by levamisole (− 8.97 kcal/mol), while phenacetin (− 8.18 kcal/mol) and caffeine (− 7.87 kcal/mol) exhibited lower, though still relevant, interaction energies.

In addition to residue-level contacts, the pharmacophoric model previously proposed for σ1R (Glennon et al. 1994) provides a complementary framework for interpreting these findings. Notably, all tested ligands except caffeine were able to satisfy the pharmacophoric requirements, in agreement with crystallographic data and reinforcing their capacity to act as functional σ1R ligands. The case of caffeine is particularly intriguing, as the molecule contains multiple nitrogen atoms that may variably satisfy the pharmacophoric constraints depending on the reference atom considered, suggesting potential alternative interaction modes that warrant further exploration. Together, these molecular docking results not only corroborate experimental evidence of σ1R engagement by cocaine but also reveal that its common adulterants may contribute to modulation of this receptor, with implications for both the pharmacological and toxicological profiles observed in real-world cocaine use.

Central to this multisystemic injury is the σ1R itself, which serves as a critical molecular scaffold and chaperone. The interaction of cocaine and its adulterants with σ1R perturbs calcium homeostasis and mitochondrial metabolism, further fueling the oxidative and apoptotic processes previously described. At the functional level, σ1R modulates ER stress, ion channels (such as Kv1.2 and NMDA), and dopamine receptor interactions, which collectively shape neuronal excitability and synaptic plasticity, processes central to cocaine’s reinforcing effects. Preclinical models demonstrate that inhibiting σ1R not only blunts cocaine-induced dopamine signaling and reinforcement but also reduces neurotoxic and inflammatory pathways, such as the secretion of cathepsin B in immune models (López et al. 2019).

## Conclusion

In summary, the multisystemic cytotoxicity of cocaine is driven by a progressive collapse of redox homeostasis and mitochondrial dysfunction, leading to pervasive cellular damage. Original in vitro assessments using C6 rat glioma cells demonstrate that this toxicological landscape is significantly exacerbated by the synergistic presence of common adulterants, such as levamisole, phenacetin, and caffeine. These findings indicate that adulterants identified in seized cocaine are not merely inert fillers but active enhancers of cellular injury. While the present review draws upon these controlled experimental and in silico models, the evidence suggests that the cytotoxic potential of seized cocaine may be further intensified in vivo. Factors such as chronic exposure, metabolic activation of these compounds, and their differential accumulation in target organs may amplify the deleterious effects beyond the results observed in cellular assays.

**Fig. 3:**
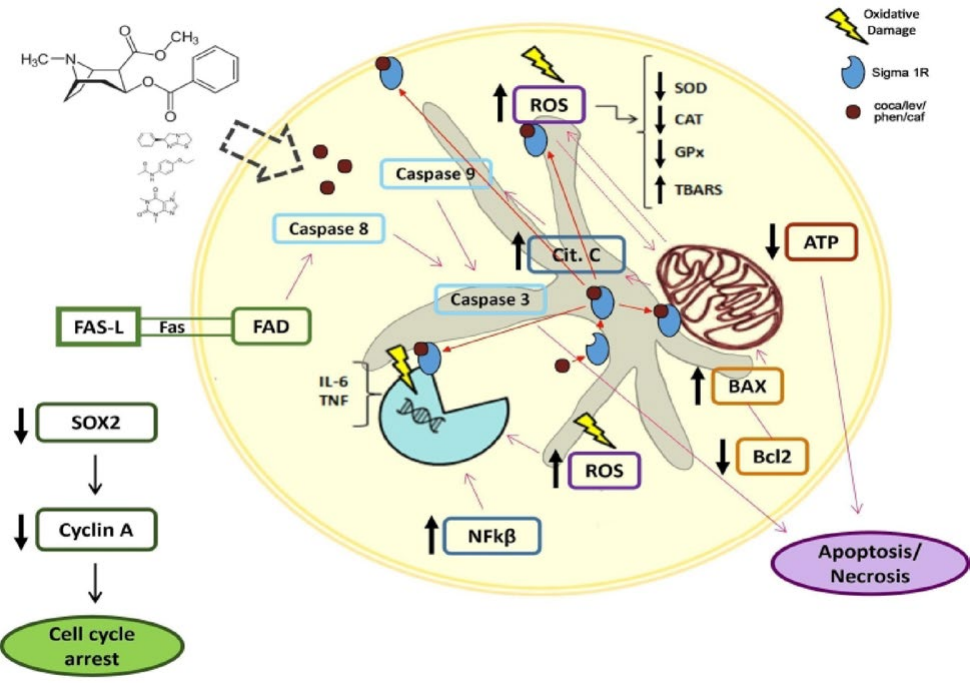
Schematic representation of cell death pathways induced by cocaine exposure and modulated by its common adulterants through σ1R interaction. Cocaine promotes oxidative stress (↑ROS), mitochondrial dysfunction (↓ATP, cytochrome c release), and activation of the intrinsic apoptotic cascade via caspases-9 and -3. These effects are accompanied by upregulation of the pro-apoptotic protein BAX, downregulation of the anti-apoptotic Bcl-2, and activation of the extrinsic pathway through FAS/FAS-L and caspase-8, leading to apoptosis or necrosis. Cocaine also triggers inflammatory signaling (IL-6, TNF, NF-κB) and downregulates SOX2 and Cyclin A, resulting in cell cycle arrest. The adulterants (levamisole, caffeine, phenacetin) are proposed to interact with σ1R, enhancing or modulating these cytotoxic effects and contributing to the overall oxidative and apoptotic response

Collectively, these mechanisms, canonical oxidative damage, adjuvant-mediated synergism, and σ1R modulation, converge to define the complex pathology of cocaine abuse, as systematically integrated in Fig. 3. Within this framework, σ1R emerges as a central molecular scaffold, mediating the transition from structural interactions to systemic injury. Future studies should prioritize in vivo investigations to validate the extent of this synergistic toxicity and to determine whether targeting σ1R can effectively mitigate the combined behavioral and cytotoxic effects of cocaine and its common adulterants. Understanding these multifaceted interactions offers a more comprehensive view of the seized cocaine reality, guiding the development of strategies to alleviate the impact of cocaine use on human health.

## Supplementary Information

Below is the link to the electronic supplementary material.

Supplementary Material 1

## Data Availability

Thein vitroandin silicodata that support the fi ndings of this review are available from the corresponding author upon request.
