# Supplementary material for: The landscape of cocaine cytotoxicity and the role of sigma-1 receptor modulation and adulterant synergism
Source: Arch Toxicol. 2026 May 13;100(8):3255–63. doi: 10.1007/s00204-026-04434-8 (PMC13379466; doi:10.1007/s00204-026-04434-8)
Supplement: Supplementary file 1 — Supplementary Material 1 [file 204_2026_4434_MOESM1_ESM.docx]

**The landscape of cocaine cytotoxicity and the role of sigma-1 receptor modulation and adulterant synergism**

Aline Steinmetz^1,2^, Carlo Frederico Moro^2,3^, Luana Freese^4^, Murilo Sander de Abreu^5^, Rodrigo Ligabue Braum^3^, Helena Maria Tannhauser Barros^4,5^, Dinara Jaqueline Moura^1,2,3*^

^1^Graduate Program in Pathology, Federal University of Health Sciences of Porto Alegre, Porto Alegre, RS, Brazil.

^2^Laboratory of Genetic Toxicology, Federal University of Health Sciences of Porto Alegre, Porto Alegre, RS, Brazil.

^3^Graduate Program in Biosciences, Federal University of Health Sciences of Porto Alegre, Porto Alegre, RS, Brazil.

^4^Laboratory of Neuropharmacology, Federal University of Health Sciences of Porto Alegre, Porto Alegre, RS, Brazil.

^5^Graduate Program in Health Sciences, Federal University of Health Sciences of Porto Alegre, Porto Alegre, Brazil.

***Corresponding author:**

Laboratory of Genetic Toxicology, Federal University of Health Sciences of Porto Alegre

Sarmento Leite 245, Sala 714, Prédio 3, Centro Histórico, Porto Alegre, RS

90050-170 Brasil

Tel.: +55 51 33039000.

E-mail [dinaram@ufcspa.edu.br](mailto:dinaram@ufcspa.edu.br)

**Supplementary Information**

**Supplementary Table**

**Table S1.** Tables showing the mean rank difference (top) and p values (bottom) for the Kruskal–Wallis test with *post-hoc* Dunn’s test referent to the viability assays of C6 cells treated with acute cocaine dose and/or contaminants. Table (**A**) shows tests with cocaine and caffeine, Table (**B**) shows tests with cocaine and phenacetin, Table (**C**) shows tests with cocaine and levamisole, and Table (**D**) shows tests with cocaine and all three contaminants. Statistically significant results (p < 0.05) are in bold. GraphPad Prism9 software (GraphPad Software, Boston, USA) was used.

| **A** | Ctrl | Coca | Caf10 | Caf20 | Caf30 | Coca +caf10 | Coca +caf20 | Coca +caf30 |
| --- | --- | --- | --- | --- | --- | --- | --- | --- |
| Ctrl | – |  |  |  |  |  |  |  |
| Coca | 11.33 | – |  |  |  |  |  |  |
|  | 0.3461 |  |  |  |  |  |  |  |
| Caf10 | 5.000 | -6.333 | – |  |  |  |  |  |
|  | >0.9999 | >0.9999 |  |  |  |  |  |  |
| Caf20 | 4.333 | -7.000 | -0.667 | – |  |  |  |  |
|  | >0.9999 | >0.9999 | >0.9999 |  |  |  |  |  |
| Caf30 | 9.333 | -2.000 | 4.333 | 5.000 | – |  |  |  |
|  | 0.7397 | >0.9999 | >0.9999 | >0.9999 |  |  |  |  |
| Coca +Caf10 | 15.67 | 4.333 | 10.67 | 11.33 | 6.333 | – |  |  |
|  | **0.0463** | >0.9999 | >0.9999 | >0.9999 | >0.9999 |  |  |  |
| Coca +Caf20 | 17.33 | 6.000 | 12.33 | 13.00 | 8.000 | 1.667 | – |  |
|  | 0.0186 | >0.9999 | 0.9103 | 0.6782 | >0.9999 | >0.9999 |  |  |
| Coca +Caf30 | 21.00 | 9.667 | 16.00 | 16.67 | 11.67 | 5.333 | 3.667 | – |
|  | **0.0019** | >0.9999 | 0.1552 | 0.1081 | >0.9999 | >0.9999 | >0.9999 |  |

| **B** | Ctrl | Coca | Fen10 | Fen20 | Fen30 | Coca  +Fen10 | Coca +Fen20 | Coca +Fen30 |
| --- | --- | --- | --- | --- | --- | --- | --- | --- |
| Ctrl | – |  |  |  |  |  |  |  |
| Coca | 15.00 | – |  |  |  |  |  |  |
|  | 0.2608 |  |  |  |  |  |  |  |
| Fen10 | 4.333 | -10.67 | – |  |  |  |  |  |
|  | >0.9999 | >0.9999 |  |  |  |  |  |  |
| Fen20 | 5.667 | -9.33 | 1.333 | – |  |  |  |  |
|  | >0.9999 | >0.9999 | >0.9999 |  |  |  |  |  |
| Fen30 | 8.667 | -6.33 | 4.333 | 3.000 | – |  |  |  |
|  | >0.9999 | >0.9999 | >0.9999 | >0.9999 |  |  |  |  |
| Coca +Fen10 | 16.00 | 1.000 | 11.67 | 10.33 | 7.333 | – |  |  |
|  | 0.1552 | >0.9999 | >0.9999 | >0.9999 | >0.9999 |  |  |  |
| Coca +Fen20 | 14.33 | -0.667 | 10.00 | 8.667 | 5.667 | -1.667 | – |  |
|  | 0.3630 | >0.9999 | >0.9999 | >0.9999 | >0.9999 | >0.9999 |  |  |
| Coca +Fen30 | 20.00 | 5.000 | 15.67 | 14.33 | 11.33 | 4.000 | 5.667 | – |
|  | **0.0147** | >0.9999 | 0.1851 | 0.3630 | >0.9999 | >0.9999 | >0.9999 |  |

| **C** | Ctrl | Coca | Lev10 | Lev20 | Lev30 | Coca +Lev10 | Coca +Lev20 | Coca +Lev30 |
| --- | --- | --- | --- | --- | --- | --- | --- | --- |
| Ctrl | – |  |  |  |  |  |  |  |
| Coca | 13.00 | – |  |  |  |  |  |  |
|  | 0.6773 |  |  |  |  |  |  |  |
| Lev10 | 2.333 | -10.67 | – |  |  |  |  |  |
|  | >0.9999 | >0.9999 |  |  |  |  |  |  |
| Lev20 | 5.000 | -8.00 | 2.667 | – |  |  |  |  |
|  | >0.9999 | >0.9999 | >0.9999 |  |  |  |  |  |
| Lev30 | 6.667 | -6.333 | 4.333 | 1.667 | – |  |  |  |
|  | >0.9999 | >0.9999 | >0.9999 | >0.9999 |  |  |  |  |
| Coca +Lev10 | 14.67 | 1.667 | 12.333 | 9.667 | 8.000 | – |  |  |
|  | 0.3076 | >0.9999 | 0.9093 | >0.9999 | >0.9999 |  |  |  |
| Coca +Lev20 | 15.33 | 2.333 | 13.00 | 10.333 | 8.667 | 0.667 | – |  |
|  | 0.2196 | >0.9999 | 0.6773 | >0.9999 | >0.9999 | >0.9999 |  |  |
| Coca +Lev30 | 19.00 | 6.000 | 16.667 | 14.00 | 12.33 | 4.333 | 3.667 | – |
|  | **0.0276** | >0.9999 | 0.1079 | 0.4257 | 0.9093 | >0.9999 | >0.9999 |  |

| **D** | Ctrl | Coca | Coca +3Conts |
| --- | --- | --- | --- |
| Ctrl | – |  |  |
| Coca | 3.000 | – |  |
|  | 0.5172 |  |  |
| Coca +3Conts | 6.000 | 3.000 | – |
|  | **0.0190** | 0.5172 |  |

**Supplementary Material and Methods details:**

**MTT assay**

The C6 rat glioma cell line was obtained from the American Type Culture Collection (ATCC, Rockville, MD, USA). Cultures were maintained in DMEM supplemented with 5% fetal bovine serum (FBS) at 37 °C in a humidified atmosphere containing 5% CO₂. Subculturing was performed using 0.15% trypsin–0.08% EDTA in PBS to detach adherent cells. For the experimental procedures, 5 x 10^5^ cells were plated in complete medium and allowed to stabilize for 24 h before drug exposure. Cocaine and contaminants were then added, and cells remained under treatment for an additional 24 h prior to viability analysis by MTT assay (Denizot and Lang, 1986). Briefly, after treatment cultures were rinsed with PBS and incubated for 3 h at 37 °C with 100 μL of serum-free medium containing MTT (1 mg/mL). After incubation, the medium was discarded, and the insoluble formazan product was solubilized in 200 μL of DMSO under gentle agitation for 15 min. Absorbance was recorded at 570 nm using a SpectraMax M2e microplate reader (Molecular Devices, USA). Results were expressed as a percentage relative to the untreated control, which was set at 100% viability.

**Molecular docking**

The σ1R protein structure was obtained from the RCSB PDB under id  pdb_00005hk1 (Schmidt et al. 2016) and ligand molecules were obtained from PubChem. Molecular docking calculations were carried out with DockThor v2 (de Magalhães et al. 2004; de Magalhães et al. 2014). All molecules were prepared with dedicated tools from the DockThor server. The gridboxes were defined at the known binding site, determined based on the crystallographic data and the UniProt annotation (entry (Q99720) . The box edges measured 10Å in all axes, with a discretization of 0.25Å. The standard DockThor setup was used. The resulting conformers were clustered at 2Å of RMSD. Contacting amino acid residues were assessed with PLIP (Salentin et al. 2015) and molecular representations were rendered with PyMol 1.3 (Schrödinger, Inc.).

References:

de Magalhães, C.S., et al., A dynamic niching genetic algorithm strategy for docking highly flexible ligands. Inf Sci (Ny). 2014. 289: p. 206–24. <https://doi.org/10.1016/j.ins.2014.08.002>

de Magalhães, C.S., et al.,. Selection-insertion schemes in genetic algorithms for the flexible ligand docking problem. Lect Notes Comput Sci (including Subser Lect Notes Artif Intell Lect Notes Bioinformatics). 2004. 3102: p. 368–79. <https://doi.org/10.1007/978-3-540-24854-5_38>

Denizot, F. and  Lang, R. Rapid colorimetric assay for cell growth and survival: modifications to the tetrazolium dye procedure giving improved sensitivity and reliability. J. Immunol. Meth. 89 (1986): p. 271-277

Salentin, S., et al.,. PLIP: fully automated protein-ligand interaction profiler. Nucleic Acids Res. 2015. 43(W1): W443-W447. [http://doi:10.1093/nar/gkv315](about:blank)

Schmidt, H.R., et al., Crystal structure of the human σ1 receptor. Nature. 2016. 532(7600): p. 527-530. <https://doi.org/10.1038/nature17391>
